# Simvastatin treatment boosts benefits of apoptotic cell infusion in murine lung fibrosis

**DOI:** 10.1038/cddis.2017.260

**Published:** 2017-06-08

**Authors:** Ye-JI Lee, Meung-Joo Kim, Young-So Yoon, Youn-Hee Choi, Hee-Sun Kim, Jihee Lee Kang

**Affiliations:** 1 Department of Physiology, School of Medicine, Ewha Womans University, Seoul 158-710, Korea; 2 Tissue Injury Defense Research Center, School of Medicine, Ewha Womans University, Seoul 158-710, Korea; 3 Department of Molecular medicine, School of Medicine, Ewha Womans University, Seoul 158-710, Korea

## Abstract

A single early-phase infusion of apoptotic cells can inhibit bleomycin-induced lung inflammation and fibrosis; however, it is unknown whether these effects can be enhanced with additional infusions and/or statin treatment. Here, we investigated whether an increased frequency of apoptotic cell injection, with or without efferocytosis enhancer simvastatin, facilitates therapeutic efficacy. An additional injection of apoptotic cells during the intermediate phase (7 days post-bleomycin treatment) or simvastatin administration alone on days 7–13 post-treatment did not promote anti-fibrotic responses beyond those induced by a single early apoptotic cell infusion alone. Additional administration of apoptotic cells with simvastatin further enhanced the efferocytic ability of alveolar macrophages and PPAR*γ* activity, and induced hepatocyte growth factor and interleukin-10 expression, in alveolar macrophages and lung tissue. Additional administration of apoptotic cells with simvastatin also reduced mRNA expression of bleomycin-induced epithelial-mesenchymal transition (EMT) markers in isolated alveolar type II epithelial cells, fibrotic markers in fibroblasts, and hydroxyproline in lung tissue. Enhanced anti-EMT and anti-fibrotic efficacy was confirmed by immunofluorescence and trichrome staining of lung tissue. This suggests that additional administration of apoptotic cells with simvastatin during the intermediate phase of bleomycin-induced lung fibrosis may boost the anti-fibrotic properties of early apoptotic cell infusion.

Pulmonary fibrosis is a potentially fatal disease, characterized by continuous alveolar epithelial injury and dysregulated repair, leading to myofibroblast accumulation and excessive deposition of extracellular matrix and connective tissue. Idiopathic pulmonary fibrosis (IPF) is the most common idiopathic interstitial disease of the lung and has the worst prognosis of all such diseases, with a median survival time of three to four years. Prevalence of IPF is 2–29 per 100 000 persons, with an incidence of ~10 in 100 000 persons per year, showing an upward trend.^1^ Although several drugs are currently used to treat the symptoms and slow IPF progression, no proven, efficacious treatment currently exists.

The feasibility of cellular therapy based on the immunomodulatory properties of apoptotic cells to restore or induce immune tolerance has already been evaluated in different experimental models of acute and chronic inflammation. Indeed, administration of apoptotic cells has been shown to attenuate LPS-induced acute lung injury or sepsis.^2, 3, 4^ Moreover, apoptotic cell injection has also been used to reduce the chronic phases of inflammatory arthritis,^5^ insulitis in mouse type 1 diabetes,^6^ and autoimmune inflammation.^7^ These beneficial effects have been attributed to the release of anti-inflammatory cytokines, such as transforming growth factor (TGF)-*β* and interleukin (IL)-10, by macrophages upon apoptotic cell recognition and clearance. Previously, we demonstrated that, in a murine model of pulmonary fibrosis, a single exposure to apoptotic cells 2 days post-bleomycin treatment mediates specific anti-inflammatory and anti-fibrotic effects via persistent upregulation of pro-resolving cytokines, such as IL-10, and hepatocyte growth factor (HGF), as well as cyclooxygenase (COX)-2-derived prostaglandin E2 (PGE_2_) and peroxisome proliferator-activated receptor (PPAR)*γ* activation.^8, 9, 10^ However, efficacy was only demonstrated within a narrow window of apoptotic cell application; that is, infusion at an early phase of bleomycin-induced pulmonary fibrosis was effective, but the apoptotic cells failed to ameliorate inflammatory and fibrotic responses when introduced in the intermediate or late phase of disease (7 or 14 d post-bleomycin treatment). Moreover, the therapeutic use of apoptotic cells needs to be carefully considered in cases where the capacity for apoptotic cell clearance is reduced during lung injury, as administered cells may progress into secondary necrosis, which could exacerbate inflammation or injury.^11^ Thus, the combined delivery of apoptotic cells with enhancers of efferocytosis may be required for full therapeutic efficacy, to prevent secondary apoptotic cell necrosis.

Statins are potent, cholesterol-lowering agents with broad anti-inflammatory properties.^12^ The immunomodulatory effects of statins are largely cholesterol independent. Rather, they appear to depend upon the ability of statins to post-translationally modify an extensive array of intracellular signaling molecules, including the Rho-family GTPases. Morimoto and colleagues demonstrated that lovastatin enhances clearance of apoptotic cells in the naive murine lung and *ex vivo* in alveolar macrophages from chronic obstructive pulmonary disease.^13^ Statins also display anti-inflammatory and anti-fibrotic effects in acute lung injury,^14^ bleomycin-induced pulmonary fibrosis,^15^ and inflammatory arthritis,^16^ although a direct link between these effects and phagocytosis of dying cells is not yet established. In this study, we asked whether an increased frequency of apoptotic cell injection, with or without simvastatin, an enhancer of efferocytosis, could enhance therapeutic efficacy of early-phase apoptotic cell infusion in a bleomycin-induced murine fibrosis model. We find that an additional injection of apoptotic cells in the intermediate phase (7 days post-bleomycin treatment) combined with simvastatin (20 mg/kg/d from day 7–13), following an early administration of apoptotic cells 2 days post-bleomycin treatment, further enhances efferocytic ability of alveolar macrophages and PPARγ activity. Consequently, the additional injection of apoptotic cells with a simvastatin regimen boosts the anti-epithelial–mesenchymal transition (EMT) and anti-fibrotic responses induced by early apoptotic cell infusion.

## Results

### Combined treatment with apoptotic cells and simvastatin enhances efferocytic ability of alveolar macrophages

**Figure 1:**
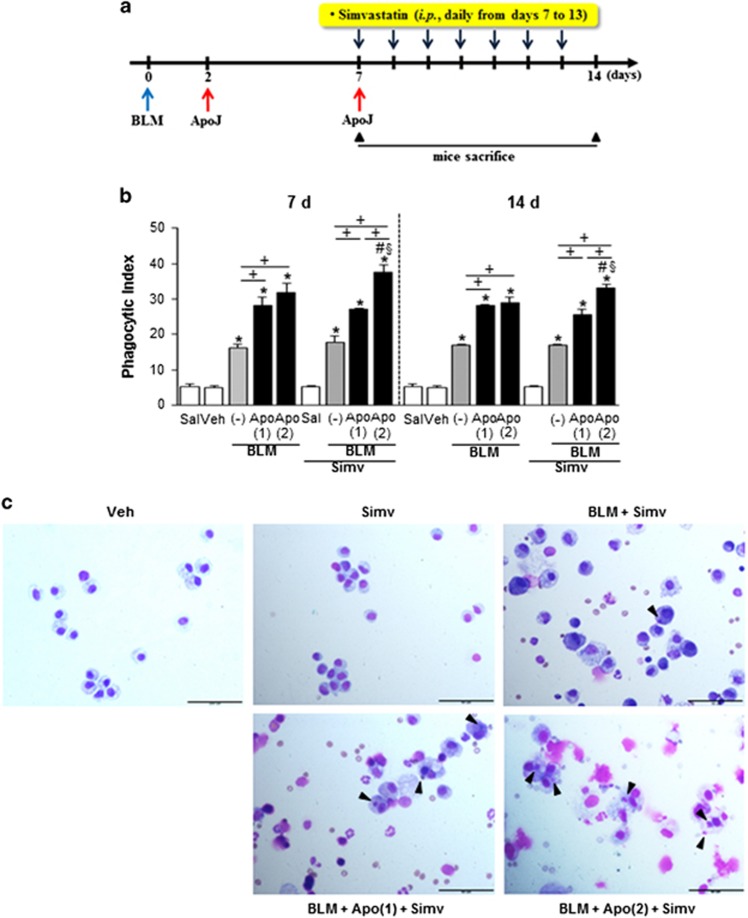
A schematic of the experimental design and effects of apoptotic cell instillation with or without simvastatin on efferocytic ability of alveolar macrophages in bleomycin-stimulated lungs. (**a**) Arrows indicate the time point of treatment with bleomycin (BLM), apoptotic Jurkat T cells (ApoJ), or simvastatin. (**b, c**) Apoptotic Jurkat cells were once instillated on day 2 or twice on days 2 and 7 after bleomycin treatment. Simvastatin (Simv; 20 mg/kg/d, i.p) or its vehicle (Veh; 2% DMSO in saline) was administered with or without second apoptotic cell instillation and every day thereafter. Mice were killed on days 7 (2 h after second apoptotic cell or simvastatin treatment) and 14 following BLM treatment. (**b**) Phagocytic indices were measured in BAL alveolar macrophages. (**c**) Representative photomicrographs from five mice per group showing cytospin-stained BAL cells on day 7 after BLM treatment. Arrowheads indicate alveolar macrophages with engulfed apoptotic cells or fragments. Scale bar=50 *μ*M. Values represent the mean±S.E.M. of results from five mice per group. *P<0.05 compared with saline control, ^+^*P*<0.05 as indicated, ^#^*P*<0.05 for BLM+twice Apo (Apo (2))+Simv *versus* BLM+single Apo (Apo (1)), ^§^*P*<0.05 for BLM+Apo (2)+Simv *versus* BLM+Apo (2)

Lovastatin increases efferocytosis in alveolar macrophages by inhibiting RhoA, affecting actin polymerization and chemotaxis.^13^ Thus, we examined whether an increased frequency of apoptotic cell injection with or without simvastatin enhances efferocytic ability of alveolar macrophages in a bleomycin-induced murine fibrosis model. The schematic drawing of experimental design was presented in the Figure 1a. We observed that an additional apoptotic cell infusion, or simvastatin treatment alone (BLM+twice Apo and BLM+single Apo+Simv, respectively) 7 days post-bleomycin treatment did not enhance phagocytic indices in alveolar macrophages 2 h after infusion, when compared to a single early apoptotic cell infusion (BLM+single Apo; Figures 1b and c). However, these indices significantly increased after an additional apoptotic cell infusion combined with simvastatin (BLM+twice Apo+Simv). Similarly, on day 14 post-bleomycin treatment, efferocytic ability of alveolar macrophages was further enhanced in the BLM+twice Apo+Simv group, compared to the BLM+Apo (single or twice) or the BLM+single Apo+Simv groups (Figure 1b).

### Combined treatment with apoptotic cells and simvastatin induces expression and activity of PPARγ

**Figure 2:**
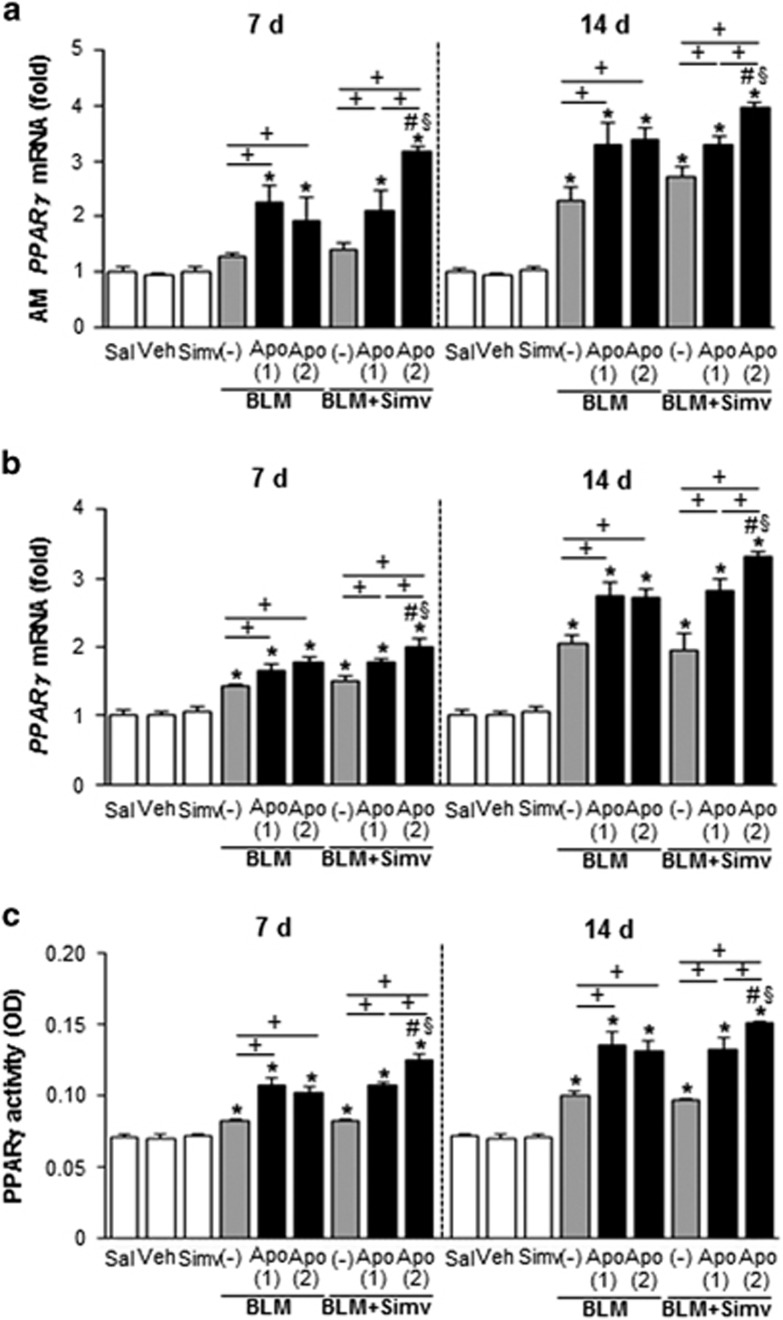
PPAR*γ* mRNA and activity induced by apoptotic cell instillation with or without simvastatin. Apoptotic Jurkat cells (Apo) were once instillated on day 2 or twice on days 2 and 7 after bleomycin (BLM) treatment. Simvastatin (Simv; 20 mg/kg/d, i.p) or its vehicle (Veh; 2% DMSO in saline) was administered with or without second apoptotic cell instillation and every day thereafter. Mice were killed on day 7 (2 h after second apoptotic cell or simvastatin treatment) and 14 following BLM treatment. PPARγ mRNA expression was analyzed by real-time PCR in (**a**) alveolar macrophages and (**b**) lung tissue. (**c**) PPARγ activity in nuclear extracts from lung tissue was analyzed as described in Materials and Methods section. Values represent the mean±S.E.M. of results from five mice per group. *P<0.05 compared with saline control, ^+^*P*<0.05 as indicated, ^#^*P*<0.05 for BLM+twice Apo (Apo (2))+Simv *versus* BLM+single Apo (Apo (1)), ^§^*P*<0.05 for BLM+Apo (2)+Simv *versus* BLM+Apo (2)

**Figure 3:**
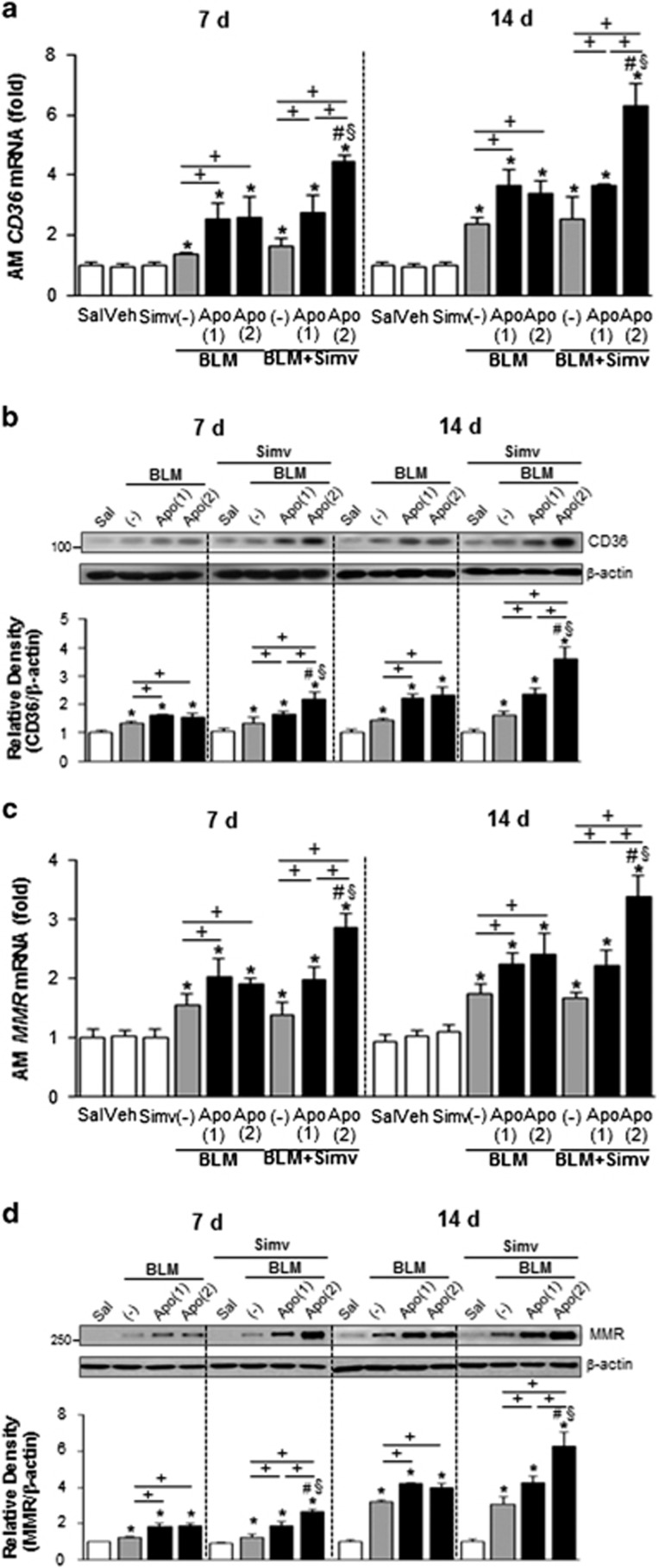
PPARγ target molecule expression induced by apoptotic cell instillation with or without simvastatin. Apoptotic Jurkat cells (Apo) were once instillated on day 2 or twice on days 2 and 7 after bleomycin (BLM) treatment. Simvastatin (Simv; 20 mg/kg/d, i.p) or its vehicle (Veh; 2% DMSO in saline) was administered with or without second apoptotic cell instillation and every day thereafter. Mice were killed on days 7 (2 h after second apoptotic cell or simvastatin treatment) and 14 following BLM treatment. (**a**) CD36 and (**c**) MMR mRNA expression was analyzed by real-time PCR in alveolar macrophages and lung tissue. (**b**, **d**) Western blot analysis of CD36 and MMR protein in lung tissue homogenates. The relative densitometric intensity was determined for each band and normalized to *α*-tubulin. Values represent the mean±S.E.M. of results from five mice per group. *P<0.05 compared with saline control, ^+^*P*<0.05 as indicated, ^#^*P*<0.05 for BLM+twice Apo (Apo (2))+Simv *versus* BLM+single Apo (Apo (1)), ^§^*P*<0.05 for BLM+Apo (2)+Simv *versus* BLM+Apo (2)

Data from our previous studies indicate that early single apoptotic cell instillation upregulates PPARγ expression and subsequent activation, leading to regulation of efferocytosis.^10^ In the present study, we examined whether an increased frequency of apoptotic cell injection, with or without simvastatin enhances PPARγ expression and its activity in alveolar macrophages and lung tissue. An additional infusion of apoptotic cells 7 days post-bleomycin treatment did not increase either the abundance of *PPAR*γ mRNA in alveolar macrophages and lung tissue or its activity in lung tissue (measured 2 h after infusion and 14 post-bleomycin treatment), as compared to the BLM+single Apo group (Figures 2a–c). However, co-administration of the second apoptotic cell infusion with simvastatin further enhanced *PPAR*γ mRNA expression in alveolar macrophages and lung tissue and activity in lung tissue, compared with the BLM+Apo (single or twice) or the BLM+single Apo+Simv groups. Similarly, mRNA and/or protein abundances of PPARγ targets, such as CD36 and macrophage mannose receptor (MMR), were enhanced in alveolar macrophages (Figures 3a and c) and lung tissue (Figures 3b and d; Supplementary Figure 1a and b) from the BLM+twice Apo+Simv group on days 7 and 14 post-bleomycin treatment. No significant changes in expression were observed in the BLM+single Apo, BLM+twice Apo, and BLM+single Apo+Simv groups. Furthermore, no statistical differences (*P*<0.05) in PPARγ mRNA expression and activity, or its target expression, were measured in the BLM+Sal and BLM+Simv groups on these days.

### Combined treatment with apoptotic cells and simvastatin induces expression of pro-resolving cytokines

**Figure 4:**
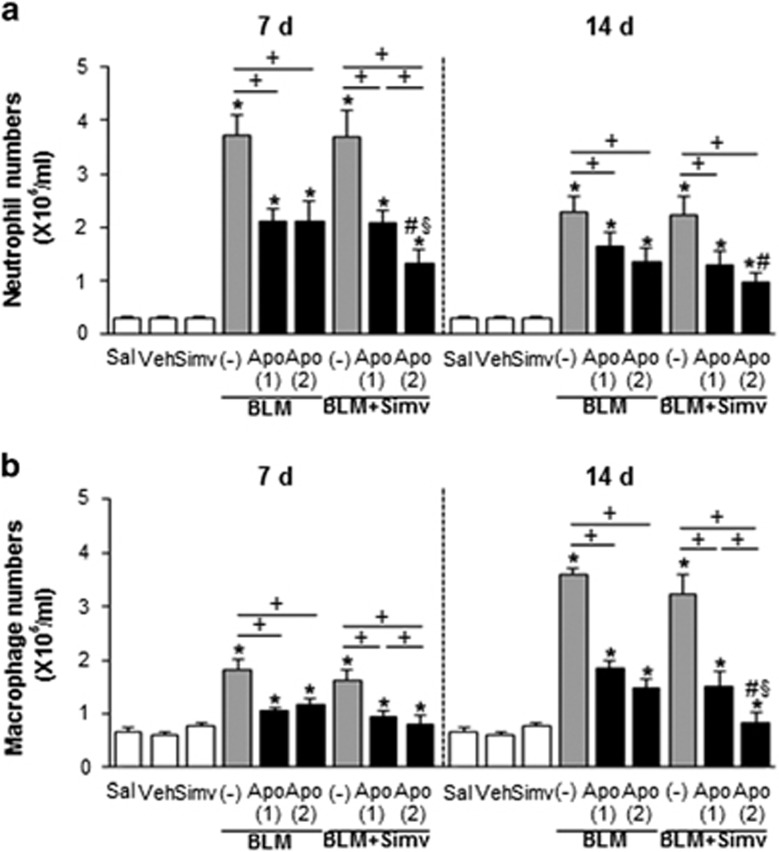
Changes in numbers of neutrophils and alveolar macrophages after apoptotic cell instillation with or without simvastatin. Apoptotic Jurkat cells (Apo) were once instillated on day 2 or twice on days 2 and 7 after bleomycin (BLM) treatment. Simvastatin (Simv; 20 mg/kg/d, i.p) or its vehicle (Veh; 2% DMSO in saline) was administered with or without second apoptotic cell instillation and every day thereafter. Mice were killed on days 7 (2 h after second apoptotic cell or simvastatin treatment) and 14 following BLM treatment. (**a**) Neutrophil and (**b**) alveolar macrophage numbers in BAL fluid. Values represent the mean±S.E.M. of results from five mice per group. *P<0.05 compared with saline control, ^+^*P*<0.05 as indicated, ^#^*P*<0.05 for BLM+twice Apo (Apo (2))+Simv *versus* BLM+single Apo (Apo (1)), ^§^*P*<0.05 for BLM+Apo (2)+Simv *versus* BLM+Apo (2)

Previously we reported that a single apoptotic cell instillation into the lungs 2 days post-bleomycin treatment exerted anti-inflammatory response and enhanced pro-resolving cytokines, including IL-10 and HGF, but reduced TGF-*β* after bleomycin treatment.^8, 9, 10^ Thus, we examined whether additional apoptotic cell instillation with or without simvastatin enhances anti-inflammatory effects by assessing numbers of neutrophils and alveolar macrophages in bronchoalveolar lavage (BAL) fluid. Co-administration of the second apoptotic cell infusion with simvastatin further reduced numbers of neutrophils at 7 and 14 days and alveolar macrophages at 14 days after bleomycin treatment, respectively, when compared to a single early apoptotic cell infusion (BLM+single Apo; Figures 4a and b). However, an additional apoptotic cell infusion or simvastatin treatment alone did not significantly affect these inflammatory cell numbers (*P*<0.05).

**Figure 5:**
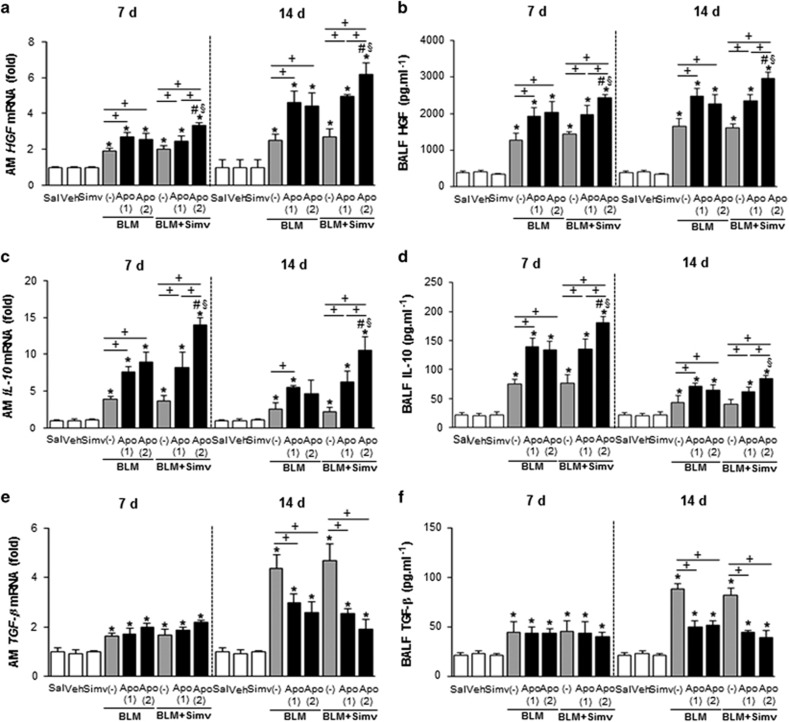
Expression of pro-resolving cytokines after apoptotic cell instillation with or without simvastatin. Apoptotic Jurkat cells (Apo) were once instillated on day 2 or twice on days 2 and 7 after bleomycin (BLM) treatment. Simvastatin (Simv; 20 mg/kg/d, i.p) or its vehicle (Veh; 2% DMSO in saline) was administered with or without second apoptotic cell instillation and every day thereafter. Mice were killed on days 7 (2 h after second apoptotic cell or simvastatin treatment) and 14 following BLM treatment. (**a**) HGF, (**c**) IL-10, and (**e**) TGF-*β*1 mRNA levels in alveolar macrophages were analyzed by quantitative real-time PCR. The levels of (**b**) HGF, (**d**) IL-10, and (**f**) TGF-*β*1 in BAL fluid were quantified by ELISA. Values represent the mean±S.E.M. of results from five mice per group. *P<0.05 compared with saline control, ^+^*P*<0.05 as indicated, ^#^*P*<0.05 for BLM+twice Apo (Apo (2))+Simv *versus* BLM+single Apo (Apo (1)), ^§^*P*<0.05 for BLM+Apo (2)+Simv *versus* BLM+Apo (2)

We also examined whether co-administration of simvastatin with apoptotic cells enhances the effects of early apoptotic cell instillation on the induction of pro-resolving cytokines. We then measured HGF and IL-10 mRNA levels and found that additional administration of apoptotic cells with simvastatin enhanced expression in alveolar macrophages (Figures 5a and c) and lung tissue (Supplementary Figure 2a and b), and upregulated the corresponding proteins in BAL fluid (Figures 5b and d), on days 7 and 14 post-bleomycin treatment, compared to the BLM+Apo (single or twice) or BLM+single Apo+Simv groups. However, levels of TGF-*β* mRNA and protein following a single apoptotic cell infusion were not further reduced by additional apoptotic cell administration, with or without simvastatin, on 14 day post-bleomycin treatment (Figures 5e and f; Supplementary Figure 2c).

### Combined treatment with apoptotic cells and simvastatin inhibits EMT and fibrotic responses

**Figure 6:**
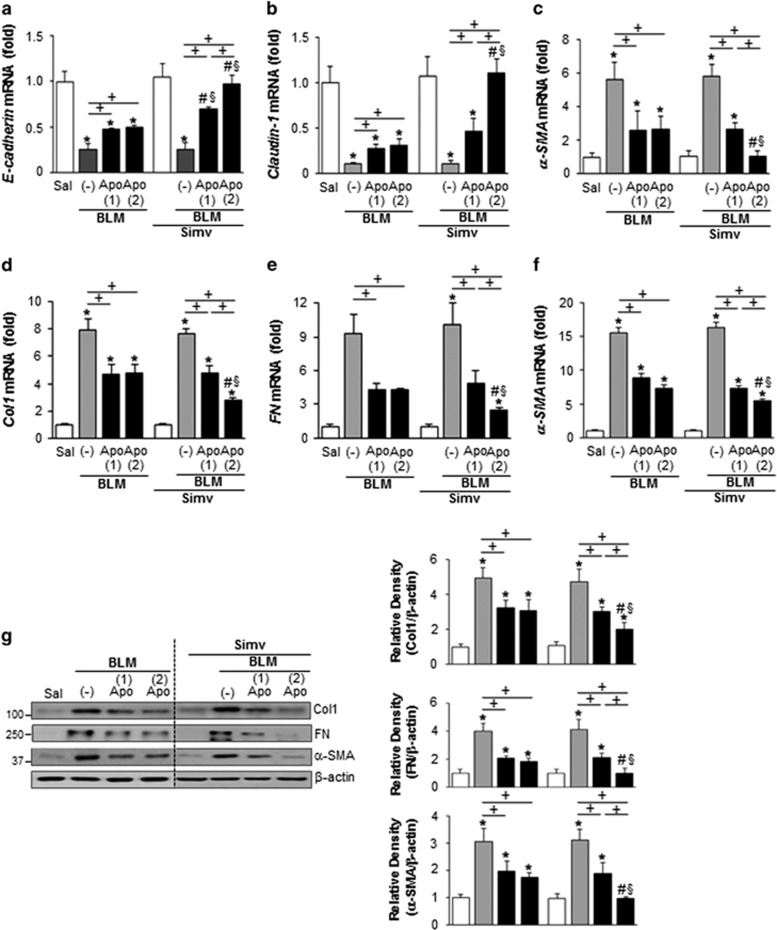
Reduction of EMT and fibrotic markers in isolated mouse alveolar type II epithelial cells and fibroblast activation by apoptotic cell instillation with or without simvastatin. Apoptotic Jurkat cells (Apo) were once instillated on day 2 or twice on days 2 and 7 after bleomycin (BLM) treatment. Simvastatin (Simv; 20 mg/kg/d, i.p.) or its vehicle (Veh; 2% DMSO in saline) was administered with or without second apoptotic cell instillation and every day thereafter. Mice were killed at 14 days after BLM treatment. (**a–c**) Primary mouse alveolar type II epithelial (AT II) cells and (**d–f**) fibroblasts were isolated from murine lungs. mRNA expression profiles of (**a**) E-cadherin, (**b**) claudin-1, and (**c**) *α-SMA* in AT II cells and (**d**) type 1 collagen *α*2 (Col1) and (**e**) fibronectin (Fn) and (**f**) *α-*SMA in fibroblasts from each group was analyzed by real-time PCR. (**g**) Homogenates of lung samples were analyzed by Western blotting to determine the relative abundances of type 1 collagen *α*2 (Col1), fibronectin (FN), and *α*-SMA in lung tissue homogenates. The relative densitometric intensity was determined for each band and normalized to *α*-actin. Values represent the mean±S.E.M. of results from five mice per group. *P<0.05 compared with saline control, ^+^*P*<0.05 as indicated, ^#^*P*<0.05 for BLM+twice Apo (Apo (2)) or single Apo (Apo (1))+Simv *versus* BLM+Apo (1), ^§^*P*<0.05 for BLM+Apo (2) or Apo (1)+Simv *versus* BLM+Apo (2)

Emerging evidence suggests that the EMT process and fibroblast activation are major events in IPF pathogenesis.^17, 18, 19^ In the present study, a second infusion of apoptotic cells with simvastatin further enhanced the mRNA abundance of several epithelial markers, such as E-cadherin and claudin-1, and reduced expression of *α-*smooth muscle actin (SMA), a marker of myofibroblast differentiation, in primary alveolar type II epithelial (AT II) cells on day 14 post-bleomycin treatment, compared to the BLM+Apo (single or twice) or BLM+single Apo+Simv (Figures 6a–c) groups. Administration of simvastatin alone following early apoptotic cell infusion also enhanced expression of these epithelial markers, but did not further reduce *α*-SMA transcript in AT II cells, compared to the BLM+Apo (single or twice) group. Moreover, additional administration of apoptotic cells with simvastatin further reduced mRNA abundance of matrix markers of fibroproliferation, such as type 1 collagen *α*2, fibronectin, and *α-*SMA in isolated fibroblasts, compared to the BLM+Apo (single or twice) or BLM+single Apo+Simv (Figures 6d–f) groups.

**Figure 7:**
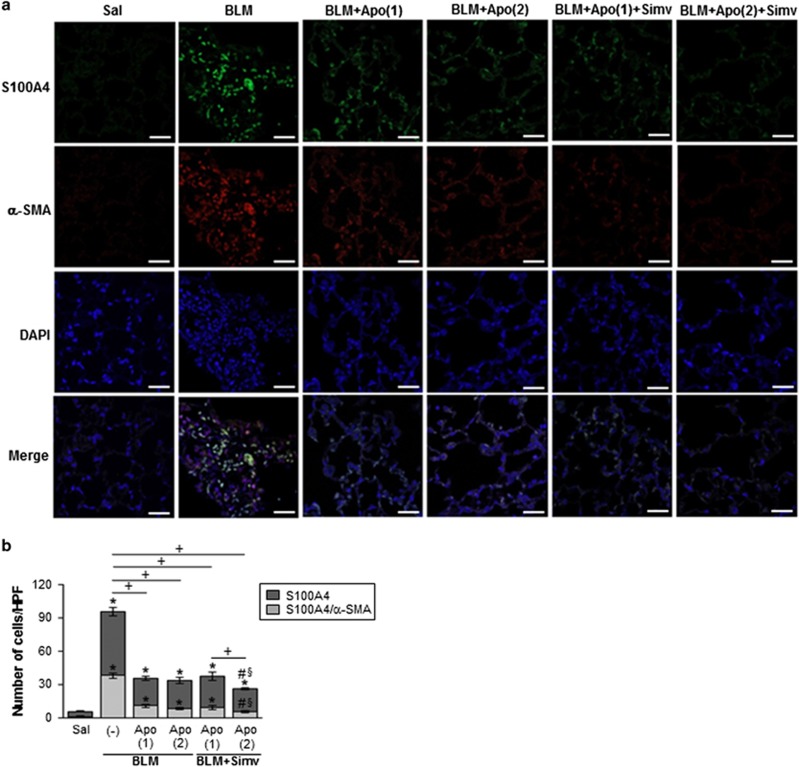
Immunofluorescence confocal microscopic findings after apoptotic cell instillation with or without simvastatin. (**a**) Immunofluorescence staining for fibroblast-specific protein-1 (S100A4, green) or *α*-SMA (red) was performed in lung sections on day 14 following BLM treatment. Arrowheads indicate co-localization of *α*-SMA in lung fibroblasts. The imaging medium was Vectashield fluorescence mounting medium containing DAPI. Scale bars=20 *μ*m. Representative images were obtained from three mice in each group. (**b**) Graph representing the number of double-positive cells of S100A4 and *α*-SMA compared with the total S100A4 positive cell population in lung parenchyma. Mean of 5 high power fields per section±S.E.M. from three mice in each group. *P<0.05 compared with saline control, ^+^*P*<0.05 as indicated, ^#^*P*<0.05 for BLM+twice Apo (Apo (2))+Simv *versus* BLM+single Apo (Apo (1)), §*P*<0.05 for BLM+Apo (2)+Simv *versus* BLM+Apo (2)

**Figure 8:**
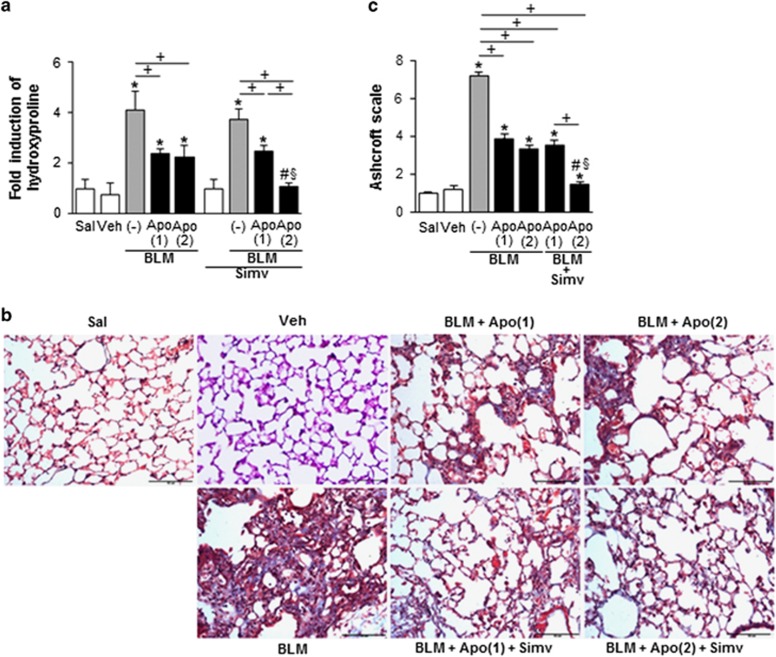
Anti-fibrotic effects of apoptotic cell instillation with or without simvastatin in bleomycin-stimulated lungs. Apoptotic Jurkat cells (Apo) were once instillated on day 2 or twice on days 2 and 7 after bleomycin (BLM) treatment. Simvastatin (Simv; 20 mg/kg/d, i.p) or its vehicle (Veh; 2% DMSO in saline) was administered with or without second apoptotic cell instillation and every day thereafter. Mice were killed at 14 days after BLM treatment. (**a**) Collagen deposition in the whole lung was determined by measuring hydroxyproline content. (**b**) Lung sections were visualized with Masson’s trichrome staining on day 21. Representative results from five mice per group are shown (scale bar=50 *μ*M). (**c**) Ashcroft scoring of the lung sections. Values represent the mean±S.E.M. of results from five mice in each group. *P<0.05 compared with saline control, ^+^*P*<0.05 as indicated, ^#^*P*<0.05 for BLM+twice Apo (Apo (2))+Simv *versus* BLM+single Apo (Apo (1)), ^§^*P*<0.05 for BLM+Apo (2)+Simv *versus* BLM+Apo (2)

Similarly, reversion of the bleomycin-induced induction of type 1 collagen *α*2, fibronectin, and *α*-SMA at the protein level in lung tissue following early apoptotic cell infusion was intensified when simvastatin was co-administered with a second apoptotic cell infusion (Figure 6g). However, a second infusion of apoptotic cells or simvastatin administration alone did not further affect expression of these markers. Double immunostaining for *α*-SMA and fibroblast-specific protein-1 (FSP1) revealed that combined therapy further enhanced the suppression of these markers observed after early apoptotic cells infusion, and the number of double-positive cells, in lung sections at 14 days post-bleomycin treatment (Figures 7a and b). Collagen accumulation in lung tissue, as determined by hydroxyproline content (Figure 8a) and Masson’s trichrome staining (Figure 8b), was also attenuated by a second infusion of apoptotic cells combined with simvastatin. Histopathological evaluation of lung fibrosis was further performed using an established Ashcroft scoring method.^20^ Fibrotic score was further decreased in the BLM+twice Apo+Simv group, compared to the BLM+Apo (single or twice) or the BLM+single Apo+Simv groups (Figure 8c). Taken together, these findings suggest that co-administration of the second apoptotic cell infusion with simvastatin further inhibits EMT process and fibroblast activation, consequently further attenuation of extracellular matrix accumulation in this bleomycin-induced murine lung fibrosis model.

## Discussion

Development of bleomycin-induced fibrosis is a complicated and dynamic process, with the local microenvironment differing at various time points after treatment. Thus, the milieu for infused apoptotic cells differs depending on the time of injection, suggesting the strategy for using apoptotic cells to prevent lung fibrosis should be carefully modulated.

In our preliminary study, we have found that administration of increased the number of apoptotic Jurkat T cells (10 × 10^7^/mouse) into the lungs 2 days post-bleomycin treatment has similar effects on mRNA or protein levels of PPAR*γ* and its target genes in lung tissue at 2 and 7 days after bleomycin treatment (data not shown). These data suggest that the number of 10 × 10^6^ apoptotic cells is efficient to prevent pulmonary inflammation and fibrosis in murine models. Here, we investigated whether additional injection of apoptotic cells, with or without simvastatin, an enhancer of efferocytosis, in the intermediate phase of disease could boost therapeutic efficacy of early apoptotic cell infusion in a bleomycin-induced murine fibrosis model. We found that additional apoptotic cell infusion 7 days post-treatment did not enhance PPARγ activity or inhibit expression of fibrotic markers in lung tissue, compared to a single early infusion of apoptotic cells. Similarly, simvastatin (20 mg/kg daily) administration alone on days 7–13 post-bleomycin treatment was unable to enhance efferocytic ability of alveolar macrophages or to inhibit bleomycin-induced fibrotic responses. Conversely, we found that co-administration of apoptotic cells on day 7 with simvastatin, and continued daily simvastatin administration, upregulates the efferocytic ability of alveolar macrophages. Specifically, alveolar macrophage phagocytic indices were immediately and gradually enhanced by additional apoptotic cell infusion with simvastatin, as compared with apoptotic cell infusion alone (single or twice), or a single early apoptotic cell infusion with subsequent simvastatin. These data suggest that simvastatin effectively acts on alveolar macrophages in response to a bolus of new apoptotic cells, but fails to enhance efferocytic ability of alveolar macrophages in the milieu with endogenously-generated apoptotic cells. Nonetheless, to determine whether ingested apoptotic cells by alveolar macrophages were exogenously administered Jurkat cells or whether they were endogenous cells, future studies using fluorescent-labeled apoptotic cells are needed.

We note that simvastatin administration (20 mg/kg daily) for 31 days, beginning 3 days before bleomycin treatment significantly attenuated bleomycin-induced increases in all parameters, including lung hydroxyproline contents, inflammatory response, fibrogenic cytokines, and profibrogenic markers in mice.^21^ However, low-dose simvastatin administration (2 mg/kg/d) from day 13 to 28 or 5 mg/kg/d for 15 days, beginning 1 day prior to bleomycin treatment had no significant effect on hydroxyproline contents,^22, 23^ indicating non-specific anti-fibrotic properties.

Enhancement of efferocytic ability following early apoptotic cell infusion may be due, in part, to activation of an alternative macrophage program associated with increased efferocytic surface receptors, including the PPARγ target molecules, CD36 and MMR.^10, 24, 25^ In our previous study,^10^ we demonstrated that co-administration of the PPARγ antagonist, GW9662, with apoptotic cells 2 days after bleomycin treatment significantly reversed the increased efferocytic surface receptors, including the PPARγ target molecules, in alveolar macrophages and lung tissue and the enhanced efferocytosis on days 2, 7 and 14 after bleomycin treatment.^10^ These data suggest that enhanced PPARγ activity plays a critical role in inducing efferocytosis by alveolar macrophages following *in vivo* exposure to apoptotic cells. Thus, persistently enhanced PPARγ activity following *in vivo* exposure to apoptotic cells might strengthen apoptotic cell recognition and clearance system and prevent a defect in the ability of macrophages to clear them during bleomycin-induced lung injury. Statins enhance efferocytosis^12, 24^ and activate PPARγ via inhibition of the RhoA-dependent signaling pathway in macrophages.^26, 27^ Here, co-administration of simvastatin further enhanced *PPAR*γ mRNA expression and activity and levels of CD36 and MMR mRNA and protein in alveolar macrophages and/or lungs on days 7 and 14 post-bleomycin treatment. This suggests the possibility that simvastatin intensifies efferocytic ability directly, via inhibition of RhoA signaling, and indirectly, via enhancement of PPARγ-dependent efferocytic surface receptors, which promotes efferocytic ability in a feed-forward manner.^10, 24^

Simvastatin treatment 7 days post-bleomycin treatment did not affect the phagocytic index and PPARγ activity in alveolar macrophages from the groups of BLM+Simva and BLM+single Apo+Simva. We do not have a clear explanation how simvastatin could not further enhance phagocytic ability of alveolar macrophages. On the other side, it has been reported that phagocytic ability of alveolar macrophages is suppressed under oxidant stress in intense lung inflammation.^28^ Notably, in human pulmonary fibrosis, there is defective efferocytic ability and an increase in the number of apoptotic cells.^29^ Under these circumstances, late simvastatin treatment (7 days post-bleomycin treatment) might not be able to affect phagocytic ability of alveolar macrophages. In studies by Morimoto and colleague, lovastatin modestly increased efferocytosis in the naive murine lung when this drug (10 mg/kg) was treated three times over 30 h before apoptotic cell instillation.^13^ On the basis of these findings, we carefully suggest that simvastatin should be initially administered before bleomycin treatment to enhance efferocytic ability of alveolar macrophages in the milieu with endogenously-generated apoptotic cells. In addition, further studies are needed to determine the alternative mechanisms, including oxidative stress, protease/anti-protease imbalance, or increase the inhibitory macrophage receptor signal regulatory protein-alpha (SIRP*α*), which all play important roles in the regulation of apoptotic cell clearance,^30, 31^ in bleomycin-induced fibrosis, using simvastatin, PPARγ agonists/antagonists, or RhoA inhibitors.

On the basis of our previous and these findings,^10^ we hypothesize that further enhancement of PPARγ activity by additional administration of apoptotic cells with simvastatin during bleomycin-induced lung injury enhances the beneficial effects of a single infusion in murine lungs. Indeed, we found that co-administration of simvastatin further enhanced transcription of pro-resolving cytokines, HGF and IL-10, compared to apoptotic cell infusion alone (single or twice) or a single apoptotic infusion with simvastatin.

Recent studies suggest that TGF-*β*1-induced EMT of alveolar epithelial cells may contribute to myofibroblast formation in murine fibrotic lungs and IPF patients.^32, 33, 34, 35, 36, 37^ We found that a second administration of apoptotic cells with simvastatin enhanced ability of an early apoptotic cell infusion to modulate expression of EMT markers,^38^ as evidenced by decreased *E-cadherin* and *claudin-1* and increased *α-SMA* transcripts in isolated AT II cells. There is accumulating data suggesting statin treatment *in vitro*^39, 40^ or *in vivo*^41^ inhibits EMT changes in various epithelial and peritoneal mesothelial cells, respectively. However, here, simvastatin alone did not affect EMT markers in AT II cells from bleomycin-treated lungs under this dosing schedule. The suppressive effects of combined treatment on fibroblast activation were also evidenced by decreased type 1 collagen *α*2, fibronectin, and *α*-SMA mRNA expression in isolated lung fibroblasts from the BLM+twice Apo+Simv group, as compared to the BLM+Apo (single or twice) or BLM+single Apo+Simv groups. Immunofluorescence staining of lung sections confirms that co-administration of simvastatin with a second apoptotic cell infusion further prevents the differentiation of lung fibroblasts in the alveolar interstitial space, as the number of *α*-SMA/FSP1 double-positive cells were further reduced in this group. In addition, further reduction in both pulmonary hydroxyproline content and collagen-stained interstitial area with damaged alveolar structures in lung sections at 14 days post-bleomycin treatment supports the observation that additional administration of apoptotic cells with simvastatin enhances anti-fibrotic effects of early apoptotic cell infusion.

Although the association between HGF and EMT has been demonstrated in various cancer models,^42, 43^ fibrotic remodeling-associated EMT in lung alveolar epithelial cells, kidney tubular epithelial cells, and peritoneal mesothelial cells is negatively modulated by HGF.^32, 44, 45, 46, 47^ In our previous study, we demonstrated that inhibition of HGF’s pathway by co-administration of the specific receptor antagonist, PHA-665752, could reverse the enhanced E-cadherin, and the reduced vimentin, FSP1 and *α*-SMA expression in lung tissue by early exposure to apoptotic cells *in vivo* on day 14 or 21 after bleomycin treatment. Taken together, our previous^38^ and these data provide *in vivo* evidence that further enhancement of HGF secretion in the BLM+twice Apo+Simv group may mediate part of the protective effect against the EMT phenotype in AT II cells and fibroblast activation in murine bleomycin-induced lung fibrosis.

Our findings demonstrate that an additional apoptotic cell infusion in the intermediate phase of bleomycin-induced disease combined with a regimen of simvastatin, following an early apoptotic cell infusion, can promote further beneficial effects on injured fibrotic lungs. This is predominantly via the enhancement of specific anti-EMT and anti-fibrotic properties in alveolar type II cells and lung fibroblasts. This co-treatment enhances efferocytic ability of alveolar macrophages and PPAR*γ*-dependent CD36 and MMR expression, leading to intensified production of pro-resolving cytokines, such as HGF and IL-10. Thus, it may promote a favorable microenvironment leading to anti-fibrotic effects via an integrated signaling network in this bleomycin-induced murine lung fibrosis model.

## Materials and Methods

### Reagents

Bleomycin, DNase I, paraformaldehyde, mouse IgG, and mouse serum were purchased from Sigma-Aldrich (St. Louis, MO, USA), and simvastatin, obtained from the Cayman Chemical Co. (Ann Arbor, MI, USA), was used as indicated.). Dispase was obtained from Corning Incorporated (Corning, NY, USA). Antibodies for western blotting were as follows: anti-CD36, anti-MMR (Cayman Chemical Co), anti-*α*- SMA, anti-fibronectin (Abcam, Cambrige, MA, USA), anti-type 1 collagen *α*2 (Santa Cruz Biotechnology, Santa Cruz, CA, USA), anti-*α*-SMA (Abcam), and anti-*β*-actin (Sigma-Aldrich).

### Animal protocols

Specific pathogen-free male C57BL/6 mice (Orient Bio, Sungnam, Korea) weighing 20–25 g were used in all experiments. The Animal Care Committee of the Ewha Medical Research Institute approved the experimental protocol. Mouse pharyngeal aspiration was used to administer the test solution containing bleomycin (5 U/kg body weight in 30 *μ*l).^8, 48^ Two days post-bleomycin treatment, saline (BLM+Sal group) or 10 × 10^6^ apoptotic Jurkat cells in 50 μl saline were administered intratracheally (*i.t.*) through pharyngeal aspiration.^2, 8, 9, 10^ A third group received an additional 10 × 10^6^ apoptotic Jurkat cells (in 50 μl saline) *i.t.* through pharyngeal aspiration on day 7 post-bleomycin treatment (BLM+twice Apo). Some of these mice simultaneously received an intraperitoneal dose of simvastatin (20 μmg/kg/d; dissolved in 2% DMSO in saline), whereas others received only the simvastatin alone, without the second apoptotic cell infusion.^21, 49, 50^ In both cases, after the initial dose, simvastatin or its vehicle only was administrated once daily. Mice were killed on day 7 (2 μh after the second apoptotic cell and/or simvastatin treatment) or on day 14 (*n*=10 per group). Treatment of mice with vehicle or simvastatin only had no effect on all parameters compared with the saline group (Figures 1–5 and 8; Supplementary Figure 3).

### BAL cells, lung tissue, and cell counts

BAL was performed through a tracheal cannula using 0.7 μml aliquots of ice-cold Ca^2+^/Mg^2+^-free phosphate-buffered medium (145 μmM NaCl, 5 μmM KCl, 1.9 μmM NaH_2_PO_4_, 9.35 μmM Na_2_HPO_4_, and 5.5 μmM dextrose; at pH 7.4) to a total of 3.5 μml for each mouse. BAL samples so obtained were centrifuged at 500 x *g* for 5 μmin at 4 °C, and cell pellets were washed and resuspended in phosphate-buffered medium. Cell counts were determined using an electronic Coulter Counter fitted with a cell sizing analyzer (Coulter Model ZBI with a channelizer 256; Coulter Electronics, Bedfordshire, UK). BAL cells were isolated and cytospun to assess phagocytic indices.^2, 8^ The phagocytic index was calculated using the following formula: (number of apoptotic bodies)/(200 total macrophages) × 100. After BAL, lungs were removed, immediately frozen in liquid nitrogen, and stored at −70°C.

### Induction of apoptosis

Human T lymphocyte Jurkat cells were obtained from the American Type Culture Collection (Rockville, MD, USA). Apoptosis was induced by UV irradiation at 254 nm for 10 min; cells were then incubated for 2 h before use. These were ~70–80% apoptotic by evaluation of nuclear morphology via light microscopy.^51^

### Isolation of alveolar macrophages

The cell pellet was washed once with PBS and resuspended in serum-free X-vivo 10 medium (Lonza, Basel, Switzerland). Suspended alveolar macrophages from mice were over 95% viable as determined by trypan blue exclusion. The cells were plated at a cell density of 5 × 10^5^ cells per well in 24-well plates, incubated for 60 min at 37 °C in 5% CO_2_, and gently washed three times with PBS to remove unattached cells.

### Isolation of murine AT II cells and lung fibroblasts

Primary murine AT II cells were isolated from mice as previously described.^52, 53^ In brief, lungs were perfused with 0.9% saline injected through the pulmonary artery until the lungs were cleared of blood. After lavage of lungs with 1 ml saline, dispase (100 units) was instilled into cleared mice lungs, the lungs were incubated for 45 min at room temperature, lung tissue was separated from large bronchi by mechanical means, and tissue transferred to a Petri dish containing Dulbecco’s modified Eagle’s medium (DMEM) with 0.01% DNase I for 10 min at 37 °C. The cells were filtered, centrifuged, and resuspended for sequential plating on mouse IgG (0.75 mg/ml)–coated Petri dishes followed by cell culture dishes, each at 37 °C for 1 h, in order to remove macrophages and fibroblasts, respectively. The final cell isolates were seeded on Type I collagen-coated 35-mm dishes in Ham’s F12 culture medium supplemented with 15 mM HEPES, 0.8 mM CaCl2, 0.25% BSA, 5 mg/ml insulin, 5 mg/ml transferrin, 5 ng/ml sodium selenite, and 2% mouse serum. It has been reported that the isolated type II cells were ~90 % pure, as assessed using cytokeratin staining and Nile red-positive vacuoles and pro-SP-C immunofluorescence stain.^53, 54^ For confocal microscopy confirmation, after fixation and permeabilization, cells were stained with rabbit polyclonal pro-SP-C Ab (1:200; Abcam, cat# ab40879). Subsequently, cells were incubated with Alexa Fluor 488-conjugated goat anti-rabbit IgG (Molecular Probes, Life Technologies, Carlsbad, CA, USA). The slides were mounted with VECTASHIELD Mounting Medium with DAPI (Vector Laboratories, Burlingame, CA, USA). The slides were imaged using a confocal microscope (LSM5 PASCAL; Carl Zeiss, Jena, Germany; Supplementary Figure 4).

Primary murine lung fibroblasts were isolated from mice and purified using a modification of published methods.^55^ Briefly, mouse lungs were cut into small pieces, minced, and digested enzymatically by DNase I in DMEM with 5% FBS for 90 min. After filtration (pore size 100 and 40 μm; SPL Life Sciences (Pocheon-si, Korea, cells were centrifuged, washed, and cultured in 6-cm dishes in DMEM medium containing 10% FBS for 3 days. Confluent cells at first passage were used for mRNA analysis.

### Quantitative real-time PCR

Gene expression was analyzed using quantitative real-time (qRT)–PCR on a StepOnePlus system (Applied Biosystems, Life Technologies, Carlsbad, CA, USA). A total of 50 ng cDNA was used for each assay. PCR Primer sets (supplementary table S1) were designed using Primer Express software. All data were normalized hypoxanthine-guanine phosphoribosyltransferase (HPRT)^56^ and are reported as fold change in expression relative to control.

### Western blot analysis

Lung tissue homogenates were separated on 10% SDS-polyacrylamide gels and electrophoretically transferred onto nitrocellulose paper. Membranes were blocked at room temperature with Tris-buffered saline containing 5% skin milk and then incubated at room temperature with various anti-mouse primary antibodies and probed with mouse anti-mouse or anti-rabbit HRP-conjugated secondary antibody. Bands were visualized using enhanced chemiluminescence.

### Measurement of PPAR*γ* activity

PPARγ activity was determined in nuclear extracts (8 *μ*g) from lung tissue prepared using a TransAM PPAR*γ* Transcription Factor Assay kit (Active Motif, Carlsbad, CA, USA), according to manufacturer instructions.

### Enzyme-linked Immunosorbent assay (ELISA)

BAL fluid samples were assayed with ELISA kits for HGF, IL-10, and TGF-*β*, according to manufacturer instructions (R&D Systems, Minneapolis, MN, USA).

### Measurement of hydroxyproline

Lung hydroxyproline content was measured using a hydroxyproline assay kit (Nanjing Jiancheng Bioengineering Institute, Nanjing, China), according to manufacturer instructions.

### Immunohistochemistry

Sections (4 *μ*m) were obtained from formalin-fixed, paraffin-embedded tissues. Slides were deparaffinised twice in xylene and rehydrated through graded ethanol solutions into distilled water. Masson’s trichrome staining was used to evaluate collagen deposition. Fibrosis was quantified using the entire lung by the Ashcroft scoring system.^20^ The degree of fibrosis was graded from 0 (normal lung) to 8 (total fibrosis). The mean score from all fields at × 200 magnification (more than 20 fields/lung section) was taken as the fibrosis score. For immunofluorescence analysis, sections were incubated with primary antibodies against *α*-SMA, FSP1, or control rabbit IgG at room temperature. The sections were incubated with Texas Red-conjugated anti-mouse IgG and FITC-conjugated goat anti-rat IgG (Vector Laboratories, Inc). Sections were washed with Tris-buffered saline between all steps and then mounted in Vectashield Mounting Medium with DAPI. All slides were imaged using a confocal microscope.

### Statistical analysis

Values are expressed as the mean±S.E.M. Analysis of variance was applied for multiple comparisons, and Tukey’s *post hoc* test was applied where appropriate. Student’s *t-*tests were used for comparisons of two sample means. A *P*-value <0.05 was considered statistically significant. All data were analyzed using JMP software (SAS Institute, Cary, NC, USA).
