# Supplementary material for: Simvastatin treatment boosts benefits of apoptotic cell infusion in murine lung fibrosis
Source: Cell Death Dis. 2017 Jun 8;8(6):e2860–. doi: 10.1038/cddis.2017.260 (PMC5520916; doi:10.1038/cddis.2017.260)
Supplement: Supplementary Information [file cddis2017260x1.docx]

# Simvastatin treatment boosts benefits of apoptotic cell infusion in murine lung fibrosis

Ye-JI Lee, Meung-Joo Kim, Young-So Yoon, Youn-Hee Choi, Hee-Sun Kim and Jihee Lee Kang

**Supplementary Table 1.** Primer sequences used in real-time qPCR

| **Target gene**  **(Mouse)** | **Primer** | **Sequences (5’ to 3’)** |
| --- | --- | --- |
| ***PPARγ*** | forward | GCCCTTTGGTGACTTTATGG |
|  | reverse | CAGCAGGTTGTCTTGGATGT |
| ***CD36*** | forward | TTGTACCTATACTGTGGCTAAATGAGA |
|  | reverse | CTTGTGTTTTGAACATTTCTGCTT |
| ***MMR*** | forward | AGAAAATGCACAAGAGCAAGC |
|  | reverse | GGAACATGTGTTCTGCGTTG |
| ***HGF*** | forward | CACCCCTTGGGAGTATTGTG |
|  | reverse | GGGACATCAGTCTCATTCACAG |
| ***IL-10*** | forward | GCTCTTACTGACTGGCATGAG |
|  | reverse | CGCAGCTCTAGGAGCATGTG |
| ***TGF-β1*** | forward | TGGAGCAACATGTGGAACTC |
|  | reverse | TGCCGTACAACTCCAGTGAC |
| ***COL1A-2***  ***(Collagen,type1,alpha2)*** | forward | CAAGAAGACATCCCTGAAGTC |
|  | reverse | ACAGTCCAGTTCTTCATTGC |
| ***Fibronectin*** | forward | CACGATGCGGGTCACTTG |
|  | reverse | CTGCAACGTCCTCCTCATTCTTC |
| ***E-cadherin*** | forward | GCACTCTTCTCCTGGTCCTG |
|  | reverse | TATGAGGCTGTGGGTTCCTC |
| ***CLDN1***  ***(Claudin-1)*** | forward | ATGCCAATTACCATCAAGGC |
|  | reverse | AGCACCGGGCAGATACAGT |
| ***α-SMA*** | forward | CCACCGCAAATGCTTCTAAGT |
|  | reverse | GGCAGGAATGATTTGGAAAGG |
| ***HPRT*** | forward | CCAGTGTCAATTATATCTTCAAC |
|  | reverse | CAGACTGAAGAGCTACTGTAATG |

**
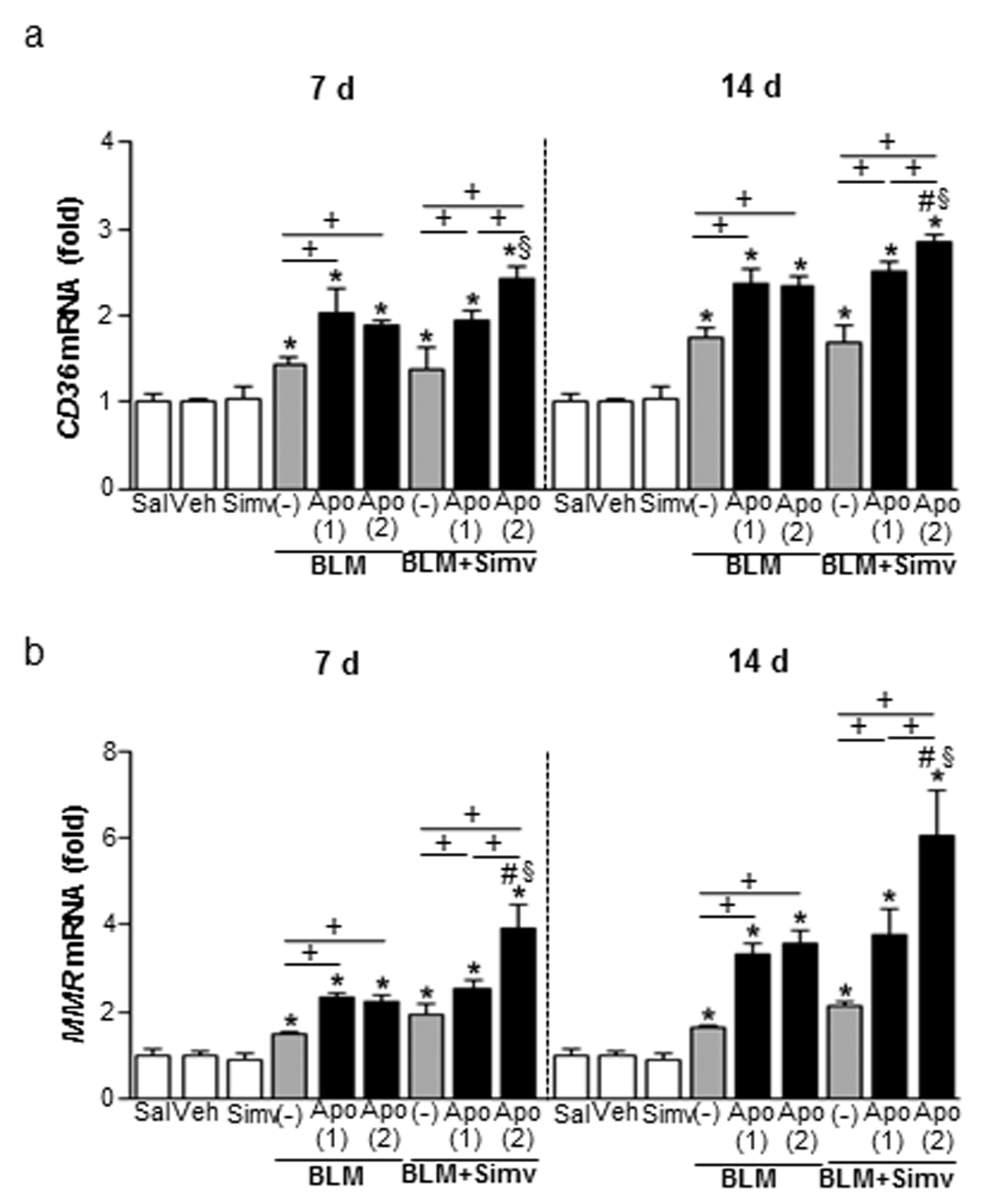
**

**Supplementary Figure 1. *CD36* and *MMR* mRNA expression in lug tissue induced by apoptotic cell instillation with or without simvastatin. A**poptotic Jurkat cells (Apo) were once instillated on day 2 or twice on days 2 and 7 after bleomycin (BLM) treatment. Simvastatin (Simv; 20 mg/kg/d, i.p) or its vehicle (Veh; 2% DMSO in saline) was administered with or without second apoptotic cell instillation and every day thereafter. Mice were euthanized on days 7 (2 h after second apoptotic cell or simvastatin treatment) and 14 following BLM treatment. (a) *CD36* and (b) *MMR* mRNA expression was analyzed by real-time PCR in lung tissue. Values represent the mean ± SEM of results from five mice per group. *: p < 0.05 compared with saline control, ^+^: p < 0.05 as indicated, ^#^: p<0.05 for BLM + twice Apo (Apo (2)) + Simv versus BLM + single Apo (Apo (1)), ^§^: p<0.05 for BLM + Apo (2) + Simv versus BLM + Apo (2).

**
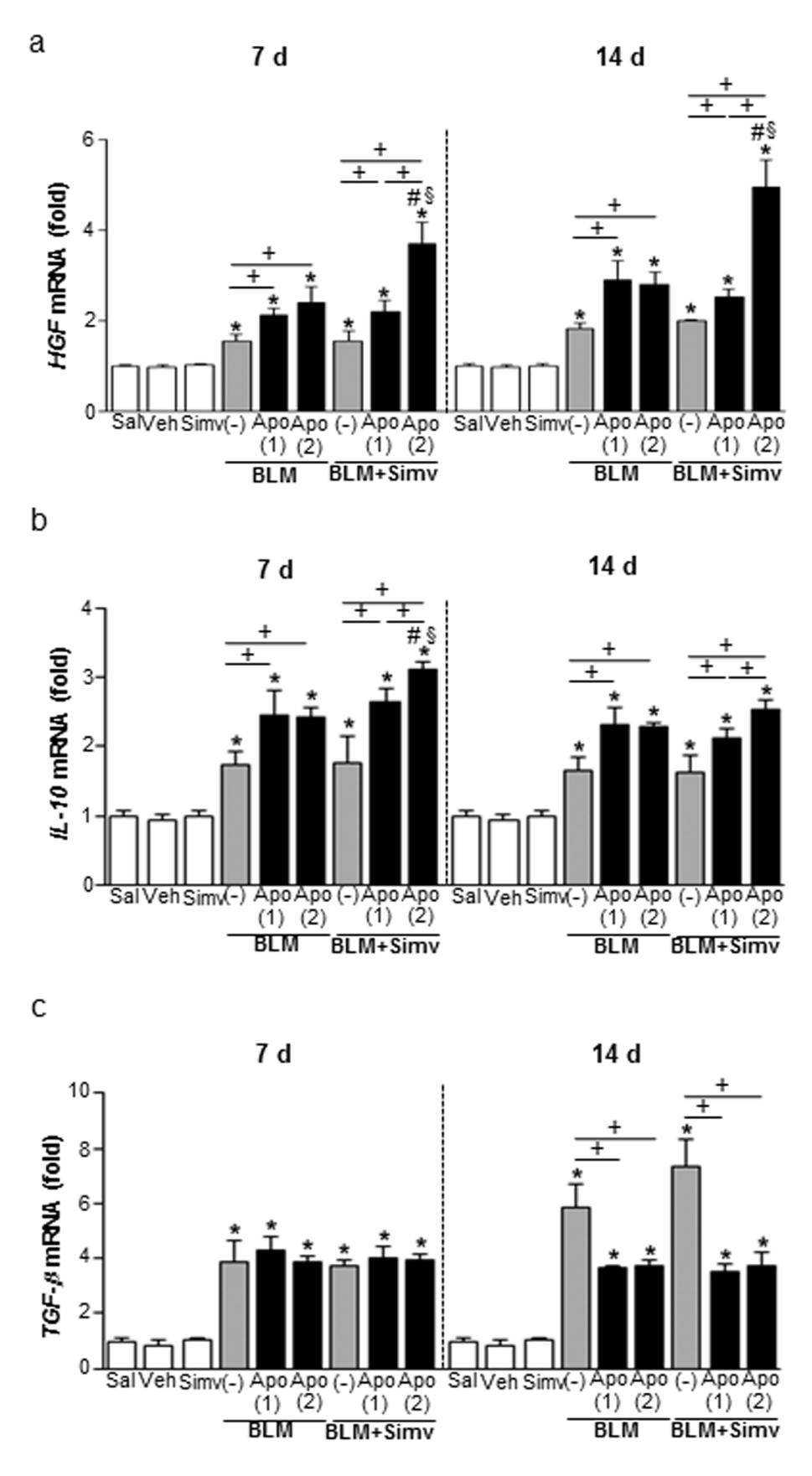
**

**Supplementary Figure 2. mRNA expression of pro-resolving cytokines in lung tissue after apoptotic cell instillation with or without simvastatin.** Apoptotic Jurkat cells (Apo) were once instillated on day 2 or twice on days 2 and 7 after bleomycin (BLM) treatment. Simvastatin (Simv; 20 mg/kg/d, i.p) or its vehicle (Veh; 2% DMSO in saline) was administered with or without second apoptotic cell instillation and every day thereafter. Mice were euthanized on days 7 (2 h after second apoptotic cell or simvastatin treatment) and 14 following BLM treatment. (a) HGF, (b) IL-10, and (c) TGF-β1 mRNA levels in lung tissue were analyzed by quantitative real time-PCR. Values represent the mean ± SEM of results from five mice per group. *: p < 0.05 compared with saline control, ^+^: p < 0.05 as indicated, ^#^: p<0.05 for BLM + twice Apo (Apo (2)) + Simv versus BLM + single Apo (Apo (1)), ^§^: p<0.05 for BLM + Apo (2) + Simv versus BLM + Apo (2).

**
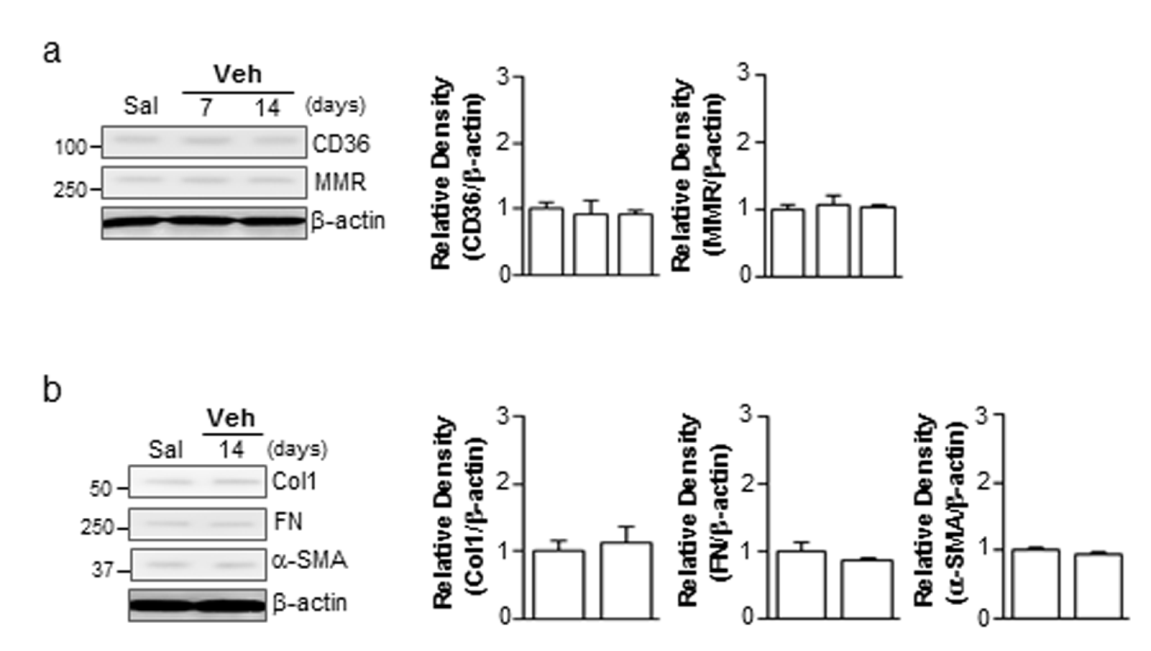
**

**Supplementary Figure 3. PPARγ target molecule and fibrotic marker expression in lug tissue after vehicle treatment.** After intratracheal instillation of saline alone (Sal), vehicle (Veh; 20% DMSO in saline, i.p.) was was administered 7 days after bleomycin treatment and every day thereafter. Mice were euthanized at 2 h after Veh treatment and 14 days after saline treatment. Homogenates of lung samples were analyzed by Western blotting to determine the relative abundances of CD36, MMR, type 1 collagen α2 (Col1), fibronectin (FN), α-SMA, and β-actin in lung tissue homogenates. The relative densitometric intensity was determined for each band and normalized to β-actin. Values represent the mean ± SEM of results from five mice per group.


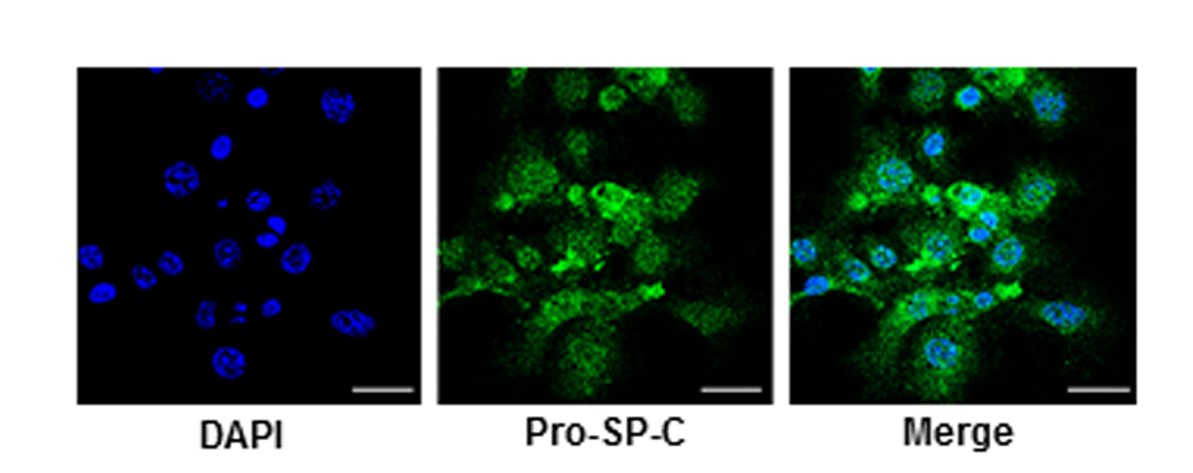


**Supplementary Figure 4. Immunofluorescence staining for pro-SP-C in primary isolated alveolar type II cells.** Immunofluorescence staining for pro-SP-C and nuclei (DAPI, blue) in the isolated alveolar type II cells from mice. Scale bars = 20 μm. Results are representative of three independent experiments.
